# Disrupted avoidance learning in functional neurological disorder: Implications for harm avoidance theories^☆^

**DOI:** 10.1016/j.nicl.2017.08.007

**Published:** 2017-08-08

**Authors:** Laurel S. Morris, Benjaman To, Kwangyeol Baek, Yee-Chien Chang-Webb, Simon Mitchell, Daniela Strelchuk, Yevheniia Mikheenko, Wendy Phillips, Michael Zandi, Allison Jenaway, Cathy Walsh, Valerie Voon

**Affiliations:** a Behavioural and Clinical Neuroscience Institute, University of Cambridge, Cambridge CB2 3EB, United Kingdom; b Department of Psychology, University of Cambridge, Cambridge CB2 3EB, United Kingdom; c Department of Psychiatry, University of Cambridge, Addenbrooke's Hospital, Cambridge CB2 0QQ, United Kingdom; d Department of Molecular Neuroscience, UCL Institute of Neurology; e Cambridgeshire and Peterborough NHS Foundation Trust, Cambridge, United Kingdom; f NIHR Cambridge Biomedical Research Centre, Cambridge, United Kingdom

**Keywords:** Functional neurological disorder, Avoidance learning, Conversion disorder, Amygdala

## Abstract

**Background:**

Functional neurological disorder (FND) is an elusive disorder characterized by unexplained neurological symptoms alongside aberrant cognitive processing and negative affect, often associated with amygdala reactivity.

**Methods:**

We examined the effect of negative conditioning on cognitive function and amygdala reactivity in 25 FND patients and 20 healthy volunteers (HV). Participants were first conditioned to stimuli paired with negative affective or neutral (CS +/CS −) information. During functional MRI, subjects then performed an instrumental associative learning task to avoid monetary losses in the context of the previously conditioned stimuli. We expected that FND patients would be better at learning to avoid losses when faced with negatively conditioned stimuli (increased harm avoidance). Multi-echo resting state fMRI was also collected from the same subjects and a robust denoising method was employed, important for removing motion and physiological artifacts.

**Results:**

FND subjects were more sensitive to the negative CS + compared to HV, demonstrated by a reinforcement learning model. Contrary to expectation, FND patients were generally more impaired at learning to avoid losses under both contexts (CS +/CS −), persisting to choose the option that resulted in a negative outcome demonstrated by both behavioural and computational analyses. FND patients showed enhanced amygdala but reduced dorsolateral prefrontal cortex responses when they received negative feedback. Patients also had increased resting state functional connectivity between these two regions.

**Conclusions:**

FND patients had impaired instrumental avoidance learning, findings that parallel previous observations of impaired action-outcome binding. FND patients further show enhanced behavioural and neural sensitivity to negative information. However, this did not translate to improved avoidance learning. Put together, our findings do not support the theory of harm avoidance in FND. We highlight a potential mechanism by which negative contexts interfere with adaptive behaviours in this under-explored disorder.

## Highlights

• Functional neurological disorder (FND) is a relatively common but elusive disorder characterized by unexplained neurological symptoms and negative affect.
• One theory of FND symptomology is related to unconscious harm avoidance, in which symptom expression occurs to avoid a stressful situation.
• We show that FND patients were more sensitive to negative information. However, this did not translate to increased harm avoidance as patients were impaired at goal-directed avoidance learning.
• This study suggests that excessive negative conditioning in this group may engender deficient, rather than increased, goal-directed cognitive ability and loss avoidance.

## 1 Introduction

Functional neurological disorder (FND), also known as conversion disorder, is characterized by unexplained neurological symptoms, including movement, seizures or sensory symptoms that are unrelated to an underlying neurological or medical disorder. It has been proposed that excessive negative affect and anxiety can exacerbate a deficient top-down regulatory system, leading to psychogenic or ‘functional’ neurological symptoms (Perez et al., 2012, Voon et al., 2010, Aybek et al., 2014, Voon et al., 2016, Carson et al., 2012). There is a relatively high prevalence of these unexplained neurological symptoms in neurology outpatient clinics (Stone et al., 2009), however we have a limited understanding of the etiology of FND. Therefore the delineation of cognitive and neural disturbances in this group is critical.

In healthy populations, learning to associate and avoid external stimuli with probable negative outcomes is vital for the selection of appropriate behaviour and environmental adaptation. Several lines of evidence suggest that FND patients are more sensitive to negative conditioning, with heightened responses to both negative affective stimuli (Bakvis et al., 2009a, Bakvis et al., 2009b) and arousal (Voon et al., 2010, Seignourel et al., 2007). Negative conditioning involves the progressive association of a neutral stimulus with fearful or negative outcomes, causing transference of negative saliency to the previously neutral stimulus (CS +). Subsequently, a fear response can be elicited from the presentation of the CS + alone, which becomes intrinsically fearful. This conditioning can be vital for rapid, automatized responses to environmental threats or danger, usurping the need for slow and deliberative cognitive processes that could hinder survival. However, FND is often characterized by a high prevalence of affective and anxiety symptoms (Bowman and Markand, 1996, Sar et al., 2004), which could be governed or exacerbated by excessive negative conditioning. Indeed, patients with psychogenic non epileptic seizures, a prevalent manifestation of FND, show increased attentional bias to negative social stimuli (angry faces) associated with higher resting cortisol levels (Bakvis et al., 2009a, Bakvis et al., 2009b, Bakvis et al., 2010). One possible theory by which FND patients express their symptoms is related to unconscious harm avoidance in which symptom expression occurs to avoid a stressful family or work situation.

Neurally, negative emotional stimuli (Aybek et al., 2014) and affective stimuli irrespective of valence (Voon et al., 2010) engender greater amygdala responses in FND patients. The amygdala largely orchestrates associative learning by linking previously neutral stimuli with representations of affective value, ascribing salience to environmental cues (Everitt et al., 2003). The basolateral amygdala mediates the initial acquisition of fear via CS-US associations (Fanselow and LeDoux, 1999, Jovanovic and Ressler, 2010) and the centromedial amygdala regulates the fear response (Jovanovic and Ressler, 2010, LeDoux, 1998) particularly in terms of physiological responses, like startle and freezing (Davis et al., 1982, LeDoux, 1992). The amygdala also plays a crucial role in the processing of emotional control and management (Cardinal et al., 2002) and via projections to the prefrontal cortex (PFC) and nucleus accumbens, the amygdala mediates motivational salience to direct goal-directed behaviour (Cardinal et al., 2002). The PFC plays an important role in mediating executive function, including goal selection, planning, anticipation and implementation (Alvarez and Emory, 2006). Specifically, the dorsolateral PFC sustains and coordinates attentional resources for goal-directed behaviour and flexibly shifts attention and valuations during learning (Rudorf and Hare, 2014, Dias et al., 1996, Arnsten and Rubia, 2012, Arnsten, 2009, Rogers et al., 2000, Hornak et al., 2004, Remijnse et al., 2005). It also importantly provides top-down regulation of attentional, inhibitory and emotional processes. In patients with FND, connectivity of the dorsolateral PFC with other motor cortical regions seems to be blunted, potentially reflecting an impairment in higher order action intention and selection (Voon et al., 2016).

Connectivity between the dorsolateral PFC and amygdala is associated with the capacity to modulate negative emotional responses with cognitive strategies (Banks et al., 2007) thereby being an expected important link in FND. However, while previous studies have demonstrated reduced connectivity between these regions in patients who have difficulty controlling affective responses (generalized social anxiety disorder (29) depression (Dannlowski et al., 2009), compulsive sexual behaviour (Schmidt et al., 2017)), there have been no studies specifically assessing the relationship between amygdala and dorsolateral PFC in FND patients during affective learning.

It is unclear how the enhancement in negative conditioning observed in FND impacts cognitive ability, and how this is expressed neurally. There is some evidence that FND patients are more likely to engage dissociative or avoidance-related strategies for coping with difficult life events, rather than planning and problem solving, or using the cognitive skills necessary to adapt to a dynamic world (Goldstein et al., 2000). Therefore, in this study, we examined the effects of negative conditioning on goal-directed avoidance learning in patients with FND. Subjects were conditioned to aversive (CS +) and neutral (CS −) stimuli. Then, during fMRI, subjects performed a goal-directed learning task to avoid losses, while being presented with the previously conditioned stimuli. We expected that FND subjects would be generally better at avoiding monetary loss (e.g. greater harm avoidance) and that the presence of the negative CS + would further enhance such avoidance learning. We also expected that FND patients would show elevated amygdala reactivity to the negative CS + and loss outcomes and that simultaneous dorsolateral PFC activity would be blunted.

## 2 Methods

### 2.1 Participants

Data were collected from 25 FND patients and 20 healthy volunteers (HV). The FND subjects were recruited both from neurologists and psychiatrists at Addenbrookes Hospital and via the FND Hope website (http://fndhope.org/). Diagnoses were made or confirmed by a neurologist from the FND clinic in Addenbrooke's Hospital, using the DSM-4 TR diagnostic criteria. All participants were screened by a psychiatrist for comorbidities and to record symptom severity. The FND patients were screened both clinically and using the Mini International Neuropsychiatric Inventory (MINI). Healthy controls were screened using the MINI. Symptom severity was rated for duration and severity (1 = Mild, limited impact on daily functioning; 2 = Moderate, noticeable impact on daily functioning with restriction of some activities; 3 = Severe, marked impact on daily functioning with restriction of activity in multiple domains. 4 = Very severe, impairment in all or virtually all domains of activity). Exclusion criteria included subjects below the age of 18, any other major neurological, current major depression greater than moderate severity, psychotic or bipolar disorder and substance use disorder. Current mild major depression and elevated depression scores with no current major depression diagnosis were allowed in the FND group. Symptoms of pain, motor (paralysis or weakness, non-epileptic seizures, tremor, chorea, tics, gait abnormalities, dystonia, myoclonus) and sensory (somatosensory, vision, hearing) functions were assessed in clinical interview that included duration and severity. All subjects were able to remain still in the scanner. HV were recruited via community-based advertisements. All participants provided written informed consent and were reimbursed for their time. The study was approved by the University of Cambridge Research Ethics Committee.

### 2.2 Task

**Fig. 1:**
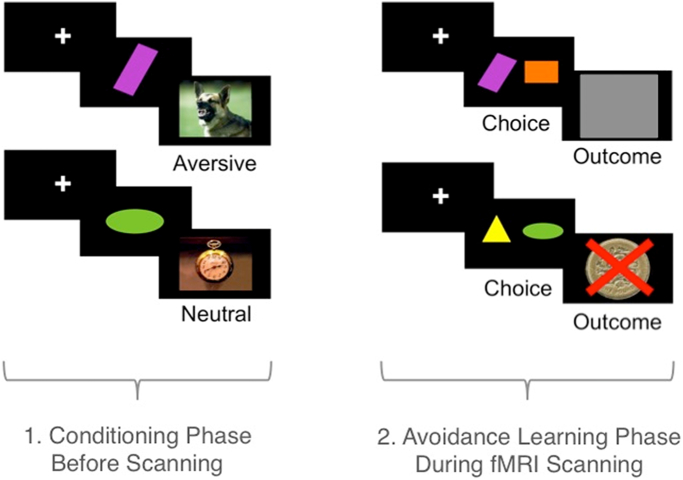
Task schematic. 1. Conditioning phase outside the scanner: One of four abstract shapes were paired with either a negative outcome (aversive sound and image) or a neutral outcome (neutral tone and image) for a total of 120 trials. Participants were separately shown a third shape with no outcome to control for familiarity. 2. Avoidance learning task during functional MRI: Following the conditioning phase, participants underwent functional MRI scanning while performing an instrumental aversion learning task with feedback. Participants chose between two stimuli (previously conditioned or familiar versus novel). The previously conditioned and familiar stimuli (CS + or CS −) was more likely to be associated with a monetary loss outcome (66/33% contingency). Subjects needed to learn to avoid the stimulus associated with loss.

See Fig. 1 for a task schematic. Outside of the scanner participants first performed a conditioning task. Subjects were seated in front of a laptop with headphones. One of four abstract shapes (cues) were paired with either a negative outcome (aversive sound and image) or a neutral outcome (neutral tone and image) for 30 trials per cue (total of 120 trials). Trials started with a 2000–4000 ms varying fixation cross, followed by the cue (1500 ms) and immediately followed by the aversive or neutral outcome (2000 ms). Participants were instructed to watch the screen and count how many times a blue frame appeared. The aversive images were rated as unpleasant images from the International Affective Pictures System (Lang et al., 2008) and paired with unpleasant sounds including high pitched screaming and nails scratching a blackboard. The neutral images were rated as neutral images from IAPS and paired with a neutral sound from a musical instrument. Separately, two new abstract images were presented both 30 times each in the absence of any outcomes, to control for effects of familiarity of stimuli and to distinguish it from any aversive or neutral information. Thus subjects were exposed to two CS +, two CS − and two familiar stimuli, equating to three conditions. Shape assignment to condition was counterbalanced between participants. Subjects used headphones throughout the conditioning trial.

Following the conditioning phase, participants underwent functional MRI scanning and performed an instrumental aversion learning task with feedback. The CS +, CS − or familiar stimuli were each paired with a novel abstract stimulus in a two-choice probabilistic learning task. The CS +, CS − or familiar stimuli were associated with a higher probability of monetary loss and the novel stimuli were associated with a lower probability of monetary loss with a 66/33% contingency (i.e. conditioned/familiar stimuli led to loss 66% of the time and no loss 33% of the time whereas the novel choice had the opposite contingency). Participants should thus learn to avoid the conditioned/familiar choice and instead choose the novel stimulus. The choice presentation phase (3000 ms) was followed by a response phase (1000 ms) in which participants made a left or right button press to indicate their response. Feedback was presented as either a £1 coin with a red cross overlaid (loss feedback) or as a grey square (no loss feedback) for 500 ms. This was followed by an inter-trial fixation cross interval varying between 1000 and 3000 ms. There were 30 trials per condition (CS +, CS −, familiar) that were randomly intermixed, resulting in 90 trials for the three conditions. This was repeated after a short break for the second set of conditioned stimuli, giving a total of 180 trials.

Outcome measures were response accuracy and trials to acquisition (trials until four consecutive correct responses). Response accuracy was also averaged across the 30 trials within conditions. Accuracy and trials to acquisition data were entered into repeated measures ANOVA testing for effects of condition (3) and group (Voon et al., 2010). Subjects were told a proportion of their losses would be subtracted from the total amounts earned across multiple tasks.

### 2.3 Q-learning

Aversion learning task data was also assessed using a standard Q-learning model (Sutton, 1998), a simple model of how the expected value of stimuli are updated based on previous associations with rewarding or punishing outcomes. In order to estimate the value assigned to the CS +, a Q-learning reinforcement learning algorithm was employed in which the initial value of the CS + was estimated as a free parameter. Q-learning (Watkins and Dayan, 1992) computes the learning signals that update internal valuations based on actual experience capturing the stimulus-action-outcome relationship. This reinforcement learning analysis finds an optimal action selection policy, ultimately providing measures of a learning rate (α) and temperature that captures noisiness or choice randomness (β). The initial value of the choice (stimulus pair) in the two-choice discrimination task is conventionally assigned a zero value representing novel stimuli in which the contingencies must be learned through experience. However, in this task adaptation, the CS + has acquired a value through prior conditioning. Thus, we added a novel parameter by assigning this initial value as a free parameter (γ) to estimate the initial value assigned to the CS + and CS −. CS +, CS − and familiar trials were modeled separately. The model estimates the expected value of a response based on individual choice-outcome sequences. This “Q value” represents the expected reward for a particular response for a given cue. The cue value is updated via the Rescorla-Wagner rule:

$$Q\left(t+1\right)=Q\left(t\right)+\alpha *\delta \left(t\right)$$

Better or worse than expected outcomes increase or decrease the value, respectively, with a prediction error of:

$$\delta \left(t\right)=R\left(t\right)-Q\left(t\right)$$

where the prediction error, δ(t), is the difference between the expected (Q(t)) and actual (R(t)) outcome. R(t) is the reinforcement given a response at trial t. The reinforcement magnitude was 0 for no loss and − 1 for loss outcomes. A softmax rule was used to estimate the probability of choosing a response given the Q value:

$$P\left(t\right)=1/\left(1+\exp\left(-Q\left(t\right)/\beta \right)\right)$$

The amplitude of value change from one trial to the next is captured by α, the learning rate. The noisiness or choice randomness is captured by β, the temperature parameter.

We also introduced a free parameter, γ, which was the estimated initial value of Q representing the CS +, CS − or familiar stimulus and was allowed to vary from − 1 to 0.

### 2.4 Functional MRI acquisition and analysis

Functional MRI data was collected during task performance at the Wolfson Brain Imaging Centre, University of Cambridge with a Siemens 3 T Tim Trio scanner and 32-channel head coil. Interleaved echo planar imaging (EPI) images were acquired (TR = 2.32 s, TE = 30 ms, 3 mm^3^ voxel size, 0.75 mm gap, FA = 78°, 64 × 64 matrix size, 39 slices) and angled 30° to the AC-PC line to avoid susceptibility signal loss in the orbitofrontal regions. Anatomical images were acquired with a T1-weighted magnetization prepared rapid gradient echo (MPRAGE) sequence (176 × 240 FOV; 1-mm in-plane resolution; inversion time, 1100 ms).

Preprocessing and data analysis was carried out using Statistical Parametric Mapping, SPM8 (http://www.fil.ion.ucl.ac.uk/spm/software/spm8/; Wellcome Department of Cognitive Neurology, London, UK). EPI data were realigned to the first image, unwarped and slice time corrected. Each individual's anatomical T1 image was co-registered with their EPI image and normalized to a standard MNI template. EPI data were smoothed using a 8 mm FWHM Gaussian kernel. Artifact Detection Tools (ART) repair toolbox for SPM (PHF et al., 2009) was used to identify and remove motion artifacts. A first level general linear model (GLM) was computed including temporal regressors for choice (including CS +, CS −, familiar), response, feedback (loss or no loss/neutral) and the fixation inter-trial interval phases. This subject-level GLM also included six rigid-body motion parameters as nuisance regressors. Between group T contrasts were computed for choice phase CS + versus CS −/familiar and separately for the CS + and CS − loss feedback phase. For comparisons between groups and correlations with behavioural performance we focused on the amygdala and dlpfc using small volume correction (SVC) assuming significance at SVC FWE *p* < 0.05.

Resting state fMRI (rsfMRI) data were acquired following the fMRI task for 10 min with eyes open. Data from 25 FND patients and 70 HV (40 women, 40.19 ± 12.70 years old) were included in this analysis, including the HV that took part in the task-based fMRI study. We have previously reported reduced inferior parietal cortex (IPC) functional connectivity with frontal regions and increased IPC connectivity with premotor and supplementary motor cortices in this FND group (Baek et al., 2017a). A multi-echo EPI sequence was used with online reconstruction (TR = 2.47 s, FA = 78°, matrix size 64 × 64, 3.75 mm in-plane resolution, FOV = 240 mm, 32 oblique slices, alternating slice acquisition, slice thickness 3.75 mm with 10% gap, iPAT factor 3, bandwidth = 1698 Hz/pixel, TE = 12, 28, 44 and 60 ms). Resting state data were analysed with multi-echo independent component analysis (ME-ICAv2.5 beta6; http://afni.nimh.nih.gov). FastICA was used to first decompose the data. Components were then categorized as BOLD or non-BOLD based on their TE-dependence as measured by the pseudo-F-statistic kappa and rho, respectively (Kundu et al., 2012). Non-BOLD components were removed by projection. Denoised functional data were coregistered with their anatomical data and normalized to an MNI template, spatially smoothed with a 6 mm FWHM Gaussian kernel, and temporally band-pass filtered between 0.008 and 0.09 Hz. Anatomical scans were segmented into grey matter, white matter and cerebrospinal fluid (CSF), and significant principal components of white matter and CSF were removed. Amygdala region of interest (ROI) with whole brain functional connectivity was computed with CONN-fMRI Functional Connectivity toolbox (Whitfield-Gabrieli and Nieto-Castañón, 2012) for SPM8. These ROI-to-voxel whole-brain connectivity maps were entered into full factorial general linear model to compare between FND patients and HV. Whole brain corrected cluster level *p* < 0.05 was considered significant.

## 3 Results

The participant characteristics have been extensively described (Baek et al., 2017b). Symptom patterns and severity scores were available for 24 FND patients (Table 1). Two participants had current depression of mild severity, 10 additional subjects had a history of depression, two had panic disorder and two had a history of obsessive-compulsive disorder. Medication use included antidepressants (*n* = 17), pregabalin (*n* = 5), gabapentin (*n* = 1), lamotrigine (*n* = 2), topiramate (*n* = 1), and a synthetic opioid (*n* = 1).

**Table 1:** Population characteristics. Reported as mean ± standard deviation (SD) (n = sample size/number of FND patients with symptom). Abbreviations: BDI-II: Beck Depression Inventory-II; df: degrees of freedom; STAI: Spielberger State-Trait Anxiety Inventory.

	FND	HV	Statistic	df	*p*-Value
Gender (F:M)	22:4	17:8	χ^2^ = 1.96	1	> 0.1
Age	42.12 ± 12.25 (*n* = 26)	40.52 ± 15.22 (*n* = 25)	*t* = 0.41	49	> 0.1
BDI-II	22.83 ± 10.64 (*n* = 24)	6.29 ± 6.0 (*n* = 21)	*U* = 56.00	43	< 0.001
STAI	45.87 ± 14.01 (*n* = 23)	37.19 ± 9.75 (*n* = 21)	*t* = 2.40	39.35	0.021
Pain	2.62 ± 0.72 (*n* = 17, 2 omissions)				
Pain type	Headache *n* = 13; Legs *n* = 3; Body *n* = 5				
Pain duration	54.50 ± 64.17 (*n* = 17, 2 omissions)				
Positive motor symptoms	2.08 ± 0.49 (*n* = 13, 2 omissions)				
Positive motor symptom type	Myoclonic jerks *n* = 2; Tremor *n* = 7; Dystonia *n* = 3; Gait abnormality *n* = 3				
Negative motor symptoms	3.15 ± 0.80 (*n* = 20, 2 omissions)				
Negative motor symptom type	Weakness: lower extremities *n* = 7, upper and lower extremities *n* = 13				
Motor symptom duration	53.51 ± 44.21 (*n* = 21, 3 omissions)				
Non-epileptic seizures	2.95 ± 1.01 (*n* = 10, 2 omissions)				
Sensory symptoms	1.97 ± 0.74 (*n* = 19, 2 omissions)				
Sensory symptom type	Loss of sensation, numbness, pins and needles *n* = 14; Tinnitus *n* = 3; Hearing loss *n* = 1; Double vision *n* = 3; Blurred vision *n* = 8; Loss *n* = 2				
Sensory symptom duration	46.79 ± 42.55 (*n* = 19, 7 omissions)				
Other symptoms	Stutter *n* = 4; Dysarthria *n* = 8; Dysphonia *n* = 4; Swallowing *n* = 8; Memory *n* = 10; Gastrointestinal *n* = 6; Genitourinary *n* = 10; Cardiovascular *n* = 5				

### 3.1 Behaviour

**Fig. 2:**
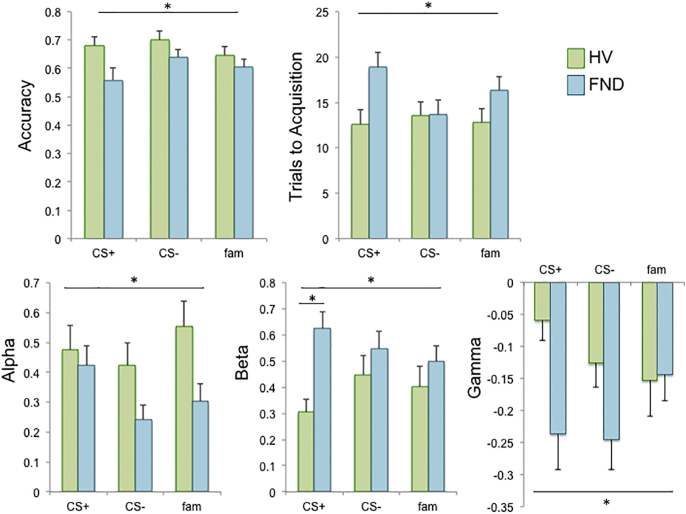
Avoidance learning captured by Q-learning reinforcement learning model. Top: Functional neurological disorder (FND) patients showed impaired avoidance learning in the presence of a negative CS +, a neutral CS − and an unconditioned familiar stimulus (group effects: accuracy, *p* = 0.019; trials to acquisition, *p* = 0.038) compared to healthy volunteers (HV). Bottom: The learning rate, alpha, was significantly lower across conditions in the FND group (*p* = 0.037). The temperature parameter, beta, was elevated in FND (*p* = 0.025), with a group × condition interaction (*p* = 0.03) and significant group difference in the CS + condition (*p* < 0.001), but not for CS − or familiar (*p* > 0.05). The initial value estimate, gamma, was also lower in FND compared to HV (*p* = 0.036).

Repeated measures ANOVA for overall avoidance learning accuracy revealed a main effect of group (F_(1,43)_ = 5.896, *p* = 0.019) of impaired accuracy in the FND group and a trend towards a main effect of condition (F_(2,42)_ = 2.571, *p* = 0.088) (Fig. 2). For trials to acquisition, there was a main effect of group (F_(1,43)_ = 4.585, *p* = 0.038) with greater trials to acquisition in the FND group and no effect of condition. There were no group × condition interaction effects (*p* > 0.05).

### 3.2 Q-learning

There was a main effect of group for α across the three conditions (F_(1,38)_ = 4.658, *p* = 0.037) with impaired learning rate in the FND group, and no condition or interaction effects (*p* > 0.05) (Fig. 2). For β, there was a main effect of group (F_(1,38)_ = 5.480, *p* = 0.025) with greater choice noisiness in the FND group and a group × condition interaction effect (F_(2,37)_ = 4.034, *p* = 0.026), and no main effect of condition (*p* > 0.05). We conducted post hoc pairwise comparisons which revealed a significant group difference in the CS + condition, (mean difference = − 0.321, 95% confidence interval = − 0.485 to − 0.156, *p* < 0.001), but not for the other two conditions (*p* > 0.05). For γ, there was a main effect of group (F_(1,38)_ = 4.748, *p* = 0.036) of a lower initial value assigned to the stimuli and a trend towards a condition × group interaction (F_(2,37)_ = 2.818, *p* = 0.073). We conducted post hoc pairwise comparisons, which revealed a significant group difference in the CS + condition (mean difference = 0.177, 95% confidence interval = 0.046–0.308, *p* = 0.010) and a trend towards a group difference in the CS − condition (mean difference = 0.118, 95% confidence interval = − 0.005–0.242, *p* = 0.060).

### 3.3 Functional MRI

**Fig. 3:**
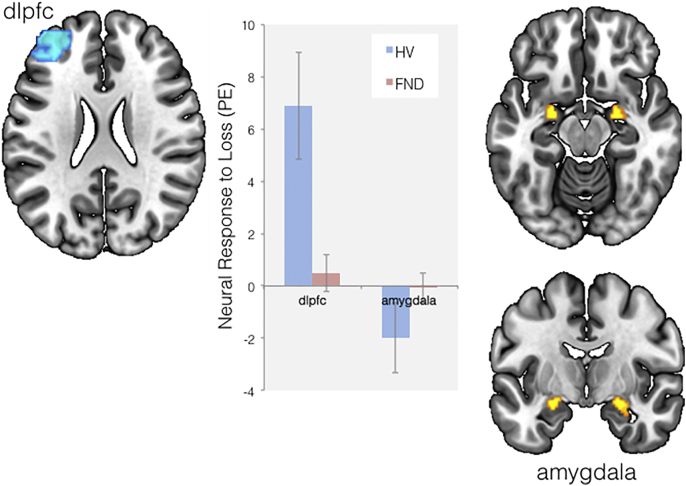
Patients with functional neurological disorder (FND) showed decreased left dorsolateral prefrontal cortex (dlpfc) and increased bilateral amygdala responses to monetary loss outcomes during a probabilistic avoidance learning task, compared to healthy volunteers (HV). Parameter estimates (PE) are plotted and peak difference clusters are displayed on a standard MNI template.

There were no between group differences in amygdala or dorsolateral PFC responses to the negative CS + during the choice presentation stage, when compared to CS − or familiar cues. When examining the feedback stage, FND subjects showed enhanced left amygdala responses to loss compared to HV (*p* = 0.048, Z = 2.83, xyz = − 22 − 6 − 14, Fig. 3), across both CS + and CS −. Furthermore, there was also a trend towards the left dorsolateral PFC having reduced neural responses to loss in FND compared to HV (*p* = 0.059, Z = 3.56, xyz = − 36 54 22, Fig. 3).

**Supplementary Fig. 1:**
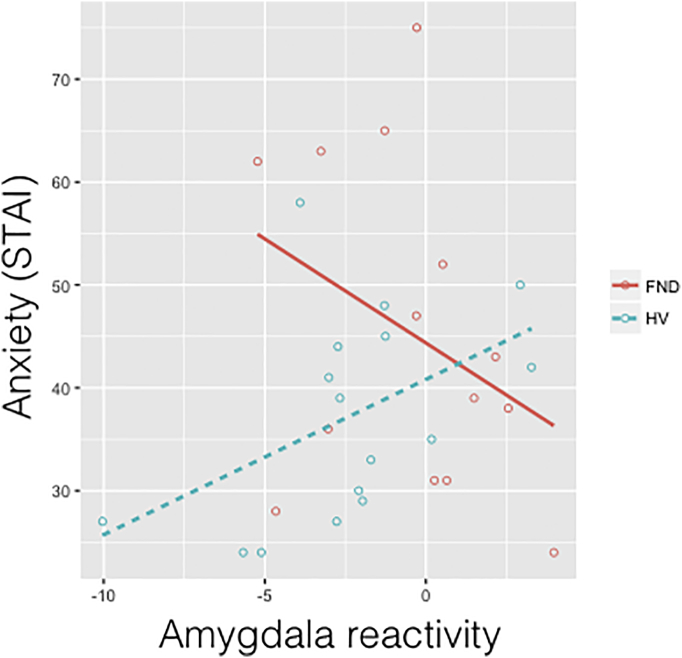
Amygdala reactivity and anxiety. Parameter estimates for amygdala responses to loss showed a significant group interaction with anxiety score (state and trait anxiety inventory, STAI), in which patients with functional neurological disorder (FND) showed a negative relationship between amygdala reactivity and anxiety but healthy volunteers (HV) showed a positive relationship.

There were no significant correlations between amygdala or dorsolateral PFC responses to loss feedback and overall performance accuracy. When examining scores of depression and anxiety, we found that for amygdala responses to loss, there was a significant STAI × group interaction (F_(1,26)_ = 5.722, *p* = 0.024) in which FND subjects showed a negative relationship between amygdala reactivity and STAI and HV showed a positive relationship (Supplementary Fig. 1), suggesting that the heightened amygdala reactivity in FND wasn't due to increased anxiety symptoms. There were no other significant relationships between depression, anxiety, symptom severity and neural responses or behavioural performance.

**Fig. 4:**
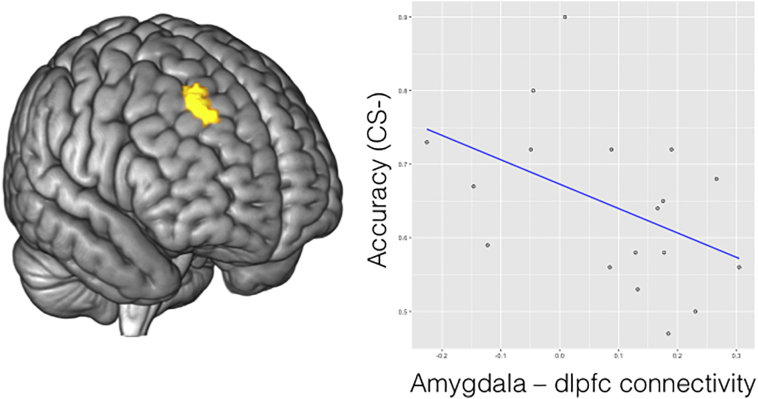
Patients with functional neurological disorder (FND) have increased resting state functional connectivity between bilateral amygdala and right dorsolateral prefrontal cortex (dlpfc, yellow cluster displayed). In the FND group, there was a trend towards negative relationship between amygdala and dlpfc connectivity with goal-directed avoidance learning accuracy in the context of a neutral CS − (*t* = − 2.03, *p* = 0.059).

Resting state functional connectivity of the bilateral amygdala and right dorsolateral PFC was increased in FND patients compared to HV (cluster level *p*(FWE) = 0.006, Cluster = 181, Z = 4.07, xyz = 27 33 51, Fig. 4). There was no significantly reduced functional connectivity of the amygdala. There were no significant correlations between amygdala and dorsolateral PFC functional connectivity and depression or anxiety scores, or amygdala or dlpfc neural responses to loss during the task. There was a trend towards a negative relationship between amygdala and dorsolateral PFC functional connectivity and accuracy in the CS − condition in FND patients (*t* = − 2.03, *p* = 0.059, Fig. 4).

## 4 Discussion

This study demonstrates two separate impairments in FND related to aversive instrumental avoidance learning and consequences of conditioned aversive stimuli. We show that FND patients have a general impairment in instrumental avoidance learning to learn the association between stimuli and outcomes (S-O) and responses and outcomes (R-O) and to use aversive goals to guide behaviours. These findings were confirmed by both behavioural and computational methods. We further show that FND patients had a more negative valuation of the aversive CS + and showed heightened amygdala but blunted dorsolateral PFC reactivity to negative outcomes.

We show that FND patients have disrupted instrumental avoidance learning in the context of both affective information (CS +/CS − stimuli) and an affectively-neutral control condition (familiar stimuli). This observation parallels a previous report of decreased implicit action-outcome binding in FND relative to healthy controls suggesting an impaired sense of agency (Kranick et al., 2013). The action-outcome binding paradigm measures the subjective temporal association between an action or response (e.g. button press) and an outcome or effect (e.g. tone). The timing of an action and outcome is subjectively contracted if the participant feels that they are the agent responsible for the outcome. In this previous study there was no specific effect of outcomes that had been conditioned to happy, neutral or fearful faces. An impaired capacity to feel that one's actions leads to an outcome may play a role in learning associations between actions and outcomes relevant to instrumental goal-directed learning. Here we show an effect with an aversive outcome of monetary losses. This finding is in opposition to the hypothesis that FND patients would demonstrate greater harm avoidance. This concept of harm avoidance suggests that symptom formation in this group has a purpose and is used to solve a problem. Proponents of this theory suggest that the functional symptom may occur unconsciously in response to an anxiety provoking situation (e.g. an intolerable familial or work situation) that then allows the patient to avoid the situation. In contrast, our findings do not support this theory and suggest FND are impaired at avoidance learning. Disputing this theory of harm avoidance might be helpful for exploring diagnostic and treatment avenues that would ultimately provide better care for the patient and reduce the stigma of the disorder.

Previous studies have demonstrated that patients with FND show diminished cognitive processing such as working memory in situations of affective processing (Bakvis et al., 2009a, Bakvis et al., 2010). Although we did not show an effect on instrumental learning, in line with these previous demonstrations of links between negative affective processing and disrupted cognitive capacity, the current work indicates that the presence of the negative CS + seems to perturb avoidance learning to a greater extent. FND patients seemed to value the negative CS + more negatively than HV, and this enhanced sensitivity was not adaptive, but was instead associated with a trend towards impaired learning and greater noise or randomness of choice behaviour.

FND patients also had enhanced amygdala and a trend towards reduced dorsolateral PFC responses to loss outcomes compared to HV. As discussed, the amygdala nuclei mediate the initial acquisition of fear via CS-US associations (Fanselow and LeDoux, 1999, Jovanovic and Ressler, 2010), regulate the fear response (Jovanovic and Ressler, 2010, LeDoux, 1998, Davis et al., 1982, LeDoux, 1992) and processes emotional control (Cardinal et al., 2002). Enhanced amygdala reactivity in the patient group is in line with evidence of greater startle response and greater amygdala reactivity to affective stimuli in FND patients (Voon et al., 2010, Seignourel et al., 2007). The amygdala is also associated with mediating cognitive process depending on attentional demands (Smith et al., 2013) and via connectivity with the dorsolateral PFC, regulates negative emotionality (Banks et al., 2007). Therefore, the disturbance in amygdala and dorsolateral PFC reactivity to negative information may suggest the neural means by which negative environmental events might impair daily activities in patients with FND. The lack of differences in amygdala or dorsolateral PFC responses to the negative CS + during the choice presentation stage might be related to their co-presentation with a novel neutral stimulus. If the negative CS + was presented alone it might elicit greater amygdala responses in the FND group.

Indeed the neutral stimulus in this study might have gained ‘safe’ properties as neutral stimuli were intermixed with fearful CS + stimuli. A similar study in healthy individuals during fMRI has demonstrated reduced amygdala responses to neutral or safe/irrelevant stimuli compared to fearful stimuli (Pollak et al., 2010). This could have been due either to reduced amygdala responses to learned safety or increased amygdala reactivity to fear. Further work is certainly needed to explore how the amygdala becomes recruited during safety learning and how it might differ in patients with FND.

Patients with unipolar depression show a similar pattern of neural responses to affective information, with increased amygdala responses to affective information (Siegle et al., 2007) that does not habituate (Siegle et al., 2002) and reduced dorsolateral PFC responses during cognitive tasks (Siegle et al., 2007). This abnormal neural response pattern can be normalized by successful antidepressant treatment (Fales et al., 2009), demonstrating that separable interacting systems are disturbed that can be targeted by pharmacological intervention. There is indeed some evidence for clinical efficacy of traditional antidepressant medication for symptoms of psychogenic non-epileptic seizures and future work should assess the extent to which these treatments can further repair these systems of affective processing and cognitive control in a broader group of FND patients. As there was no relationship between depression scores and neural reactivity, and a negative relationship between anxiety and amygdala reactivity in FND patients, the current findings may be divorced from more general effects of mood and anxiety. However, an important point to note is that anecdotally patients deny anxiety symptoms while exhibiting anxious affect and behaviour. Therefore, the current self-report measure of anxiety may be less reliable than a more objective clinical measure so conclusions should be made with caution.

The FND group also had elevated resting state functional connectivity between the amygdala and dorsolateral PFC. This was somewhat unexpected as increased resting connectivity between these regions might suggest an enhanced capacity for amygdala – dorsolateral PFC mediated affective and behavioural control. As mentioned, the dorsolateral PFC plays a regulatory role over the amygdala and voluntary emotional evaluation (Levesque et al., 2003, Stanley et al., 2008) and psychiatric groups of mood and anxiety disorders who have trouble mediating emotional responses generally show reduced connectivity between these regions (Prater et al., 2013, Dannlowski et al., 2009, Schmidt et al., 2017). However, while the PFC has been shown to dampen amygdala responses to affective information (Stanley et al., 2008), both the amygdala and dorsolateral PFC are required to maintain attentional resources during aversion learning and conditioning (Straube et al., 2007). Increased resting connectivity between these regions might therefore explain the enhanced sensitivity to the negative CS + during conditioning. This distinction in the direction of connectivity aberrancy separates FND patients from general disorders of mood and anxiety. Interestingly, increased functional connectivity between amygdala and dorsolateral PFC is found in patients with borderline personality disorder (Dudas et al., 2017), a group that is often characterized by dissociative responses to traumatic emotional events (Vermetten and Spiegel, 2014).

We had anticipated that given the enhanced neural activity to negative affective stimuli and loss feedback, the greater salience of negative stimuli might be associated with greater harm avoidance and better avoidance learning in FND patients. However, the behavioural response to enhanced salience of negative stimuli was not adaptive but interfered with goal-directed learning with greater noise in behavioural choices with aversive contexts. These findings suggest that goal-directed harm avoidance is unlikely to underlie FND symptoms. There are several plausible mechanisms that might underlie the effect of the CS + on enhanced choice randomness in FND patients. The neural network implicated in fear/negative conditioning (e.g. amygdala, insula and dorsal cingulate) overlaps with that of value representation of loss outcomes (e.g. ventromedial prefrontal cortex, ventral striatum, anterior insula, dorsal cingulate) but is not identical. Furthermore, the timing of the training and testing phases in this task differ which might induce conflict. The training phase involved a 1.5 s cue followed by the aversive stimulus lasting 2 s. The instrumental test phase involved a two-choice decision phase of 3 s followed by a 0.5 s choice and 0.5 s outcome phase. Thus, 1.5 s into the decision phase in the presence of a CS +, subjects were conditioned to expect an aversive outcome. The presence of the CS + in FND patients thus acted as a fearful context, possibly interfering with goal-directed learning and enhancing random noisy choices.

We note that the sample size is small but we further emphasize the difficulties in recruiting FND subjects for studies. A current limitation is that multiple types of presentations of this disorder have currently been merged into a single group, assuming homogeneity in behavioural, cognitive and neural dysfunction. Since the group included both positive and negative motor symptoms, with about half experiencing non-epileptic seizures, it is likely that the disorder etiology differs between subjects. As there is currently a dearth of literature investigating this group, the current study is at least a step towards examining shared deficits. This dissociation between shared and unique aberrancies is certainly a crucial goal for future studies. The FND patients were also on different medications which may impact the findings. We further emphasize that our findings demonstrate an impairment in the capacity to acquire S-O or R-O representations relevant to goal-directed learning. However, the task was not designed to dissociate goal-directed and habitual behaviours. Furthermore, to learn 3 different stimuli-pairs and their associations is more difficult; greater cognitive demands required by this task may also contribute to the observed impairments. We emphasize that this current conditioning task uses robust aversive stimuli with unpleasant imagery of high intensity and paired with negative sounds such as nails scratching on a blackboard or high pitched screaming. Thus, the capacity to elicit effects as compared to more subtle aversive stimuli may differ.

Together, we demonstrate that despite enhanced neural salience and computational value of negative stimuli in FND subjects, behavioural avoidance learning was not improved. There was no enhancement of goal-directed harm avoidance in FND patients, but rather, a generalized impairment in the capacity to use negative outcomes to guide goal-directed behaviours alongside increased amygdala reactivity to negative outcomes. Our findings suggest that learned negative or fearful environmental contexts (e.g. cues associated with a stressful event, with depressive or anxiety symptoms, or shame associated with the symptom) can impair goal-directed decision making in FND subjects. Pharmacological or behavioural interventions to enhance associative learning or to decrease the salience of negative stimuli or learned contexts may be important in FND.

The following are the supplementary data related to this article.
